# Microbiomes and Pediatric onset multiple sclerosis (MS): A systematic review

**DOI:** 10.3934/Neuroscience.2023031

**Published:** 2023-12-14

**Authors:** Sanaz Mehrabani, Mohsen Rastkar, Narges Ebrahimi, Mahsa Ghajarzadeh

**Affiliations:** 1 Associate Professor of Pediatrics Gastroenterology, Non-Communicable Pediatric Diseases Research Center, Health Research Institute, Babol University of Medical Sciences, Babol, Iran; 2 Student's Scientific Research Center, Tehran University of Medical Sciences, Tehran, Iran; 3 Multiple sclerosis research group (MSRG), Universal Scientific Education and Research Network (USERN), Tehran University of Medical Sciences, Tehran, Iran; 4 Department of Immunology, Isfahan university of medical science, Isfahan, Iran; 5 Universal Council of Epidemiology (UCE), Universal Scientific Education and Research Network (USERN), Tehran University of Medical Sciences, Tehran, Iran

**Keywords:** multiple sclerosis, microbiota, pediatrics

## Abstract

**Background:**

Gut microbiomes play a role in developing and regulating autoimmune diseases such as multiple sclerosis (MS). We designed this systematic review to summarize the evidence of the effect of gut microbiota in developing pediatric-onset MS.

**Methods:**

PubMed, Scopus, EMBASE, Web of Science, Google Scholar, references of the references and conference abstracts were comprehensively searched by two independent researchers. The search was done on January 1^st^, 2023. Data regarding the total number of patients, the name of the first author, publication year, country of origin, mean age, duration of the disease, body mass index (BMI), type of MS, Expanded Disability Status Scale (EDSS), age at disease onset and stool composition were extracted.

**Results:**

A literature search revealed 4237 published studies. After removing duplicates, we had 2045 records for evaluation. Twenty-three full texts were evaluated, and four case-control studies remained for systematic review. Three studies were conducted in the United States and one in the Netherlands. The number of participants in included studies ranged between 24 and 68. The mean age of patients at the time of study varied between 11.9 and 17.9 years, and the mean age at the onset of the disease ranged between 11.5 and 14.3 years. Most included patients were female. The results show that median richness (the number of unique taxa identified, which was provided by two studies) was higher in controls, and also Margalef index, which was reported by one study was higher in control group than the case group. The results of two studies also demonstrated that median evenness indexes (taxon distribution, Shannon, Simpson) were higher in control groups, as well as PD index (Faith's phylogenic diversity metric).

**Conclusion:**

The result of this systematic review (including four studies) showed disruption of the microbiota-immune balance in pediatric-onset MS cases.

## 1. Introduction

Multiple sclerosis (MS) is an inflammatory disease in which immune cells target cells in the central nervous system (CNS), leading to demyelination and axonal damage [1]. The exact cause is unclear. Genetics and environmental factors such as latitude, infections and stress play a role in disease development [2].

Nowadays, studies show that gut microbiota play an important role in pathogenesis of MS, as gut microbial play a role in developing and regulating autoimmune diseases [3]. Gut microbiota could stimulate pro-inflammatory T cell response in animal models of MS and in other auto-immune diseases, such as type 1 diabetes, atopic dermatitis, rheumatoid arthritis and asthma, showing the role of gut microbiota [4]–[6]. Furthermore, there is a bidirectional interaction between the gut and the CNS, known as gut-brain axis, which plays a role in developing disease [7], and there has been increasing concern regarding the role of gut microbiota in pathogenesis of MS [8].

Gut microbiota composition and risk of MS in children are somehow different from adults, as pediatrics have less exposure to risk factors of the MS during their life. Thus, evaluation of the association between gut microbiota, and the risk of developing MS in pediatric-onset MS, plays an important role in clarification of this association.

We designed this systematic review to summarize the evidence of the effect of gut microbiota in developing pediatric-onset MS.

## 2. Methods

We followed The Preferred Reporting Items for Systematic Reviews and Meta-Analyses (PRISMA) [9].

### 2.1. The inclusion criteria

We included case-control studies that reported the stool composition in patients with pediatric-onset MS.

### 2.2. The exclusion criteria were

Letters to the editor, case reports and cross-sectional studies were excluded. We also excluded studies that had no clear data regarding stool findings.

### 2.3. Information sources

PubMed, Scopus, EMBASE, Web of Science, Google Scholar, references of the references and conference abstracts were comprehensively searched by two independent researchers. The search was done on January 1^st^, 2023.

### 2.4. Search strategy

The MeSH search terms were:

((Multiple Sclerosis[MeSH Terms]) OR (Multiple Sclerosis[Text Word])) OR (Sclerosis, Multiple[Text Word])) OR (Sclerosis, Disseminated[Text Word])) OR (Disseminated Sclerosis[Text Word])) OR (Multiple Sclerosis, Acute Fulminating[Text Word])) AND (Gastrointestinal Microbiome[MeSH Terms]) OR (Microbiota[MeSH Terms])) OR (Gastrointestinal Microbio*[Text Word])) OR (Microbiome, Gastrointestinal[Text Word])) OR (Gut Microbio*[Text Word])) OR (Microbiome, Gut[Text Word])) OR (Gut Microflora[Text Word])) OR (Microflora, Gut[Text Word])) OR (Microbiota, Gut[Text Word])) OR (Gastrointestinal Flora[Text Word])) OR (Flora, Gastrointestinal[Text Word])) OR (Gut Flora[Text Word])) OR (Flora, Gut[Text Word])) OR (Microbiota, Gastrointestinal[Text Word])) OR (Gastrointestinal Microbial Communit*[Text Word])) OR (Microbial Community, Gastrointestinal[Text Word])) OR (Gastrointestinal Microflora[Text Word])) OR (Microflora, Gastrointestinal[Text Word])) OR (Gastric Microbio*[Text Word])) OR (Microbiome, Gastric[Text Word])) OR (Microbiome, Intestinal[Text Word])) OR (Intestinal Microbio*[Text Word])) OR (Microbiota, Intestinal[Text Word])) OR (Intestinal Microflora[Text Word])) OR (Microflora, Intestinal[Text Word])) OR (Intestinal Flora[Text Word])) OR (Flora, Intestinal[Text Word])) OR (Enteric Bacteria[Text Word])) OR (Bacteria, Enteric[Text Word])) OR (Microbiota*[Text Word])) OR (Microbial Communit*[Text Word])) OR (Community, Microbial[Text Word])) OR (Microbial Community Composition*[Text Word])) OR (Community Composition, Microbial[Text Word])) OR (Composition, Microbial Community[Text Word])) OR (Microbial Community Structure*[Text Word])) OR (Community Structure, Microbial[Text Word])) OR (Microbiome*[Text Word])) OR (Human Microbiome*[Text Word])) OR (Microbiome, Human[Text Word])) OR (Flora[Text Word])) OR (Microflora[Text Word])).

### 2.5. Selection process

EndNote software was used to transfer the search results to a reference manager software. Then, duplicates were deleted, and the titles/abstracts of probable studies were evaluated.

The full texts of potential studies were evaluated, data were extracted and entered in Excel data sheets. In the case of discrepancy, the third one solved the conflict.

### 2.6. Data items

Data regarding the total number of patients, first author, publication year, country of origin, mean age, duration of disease, body mass index (BMI), type of MS, Expanded Disability Status Scale (EDSS), age at disease onset and stool composition were extracted.

### 2.7. Study risk of bias assessment

The potential risk of bias of included studies was evaluated using Newcastle-Ottawa Scale (NOS) for case-control studies [10].

## 3. Results

**Figure 1.**
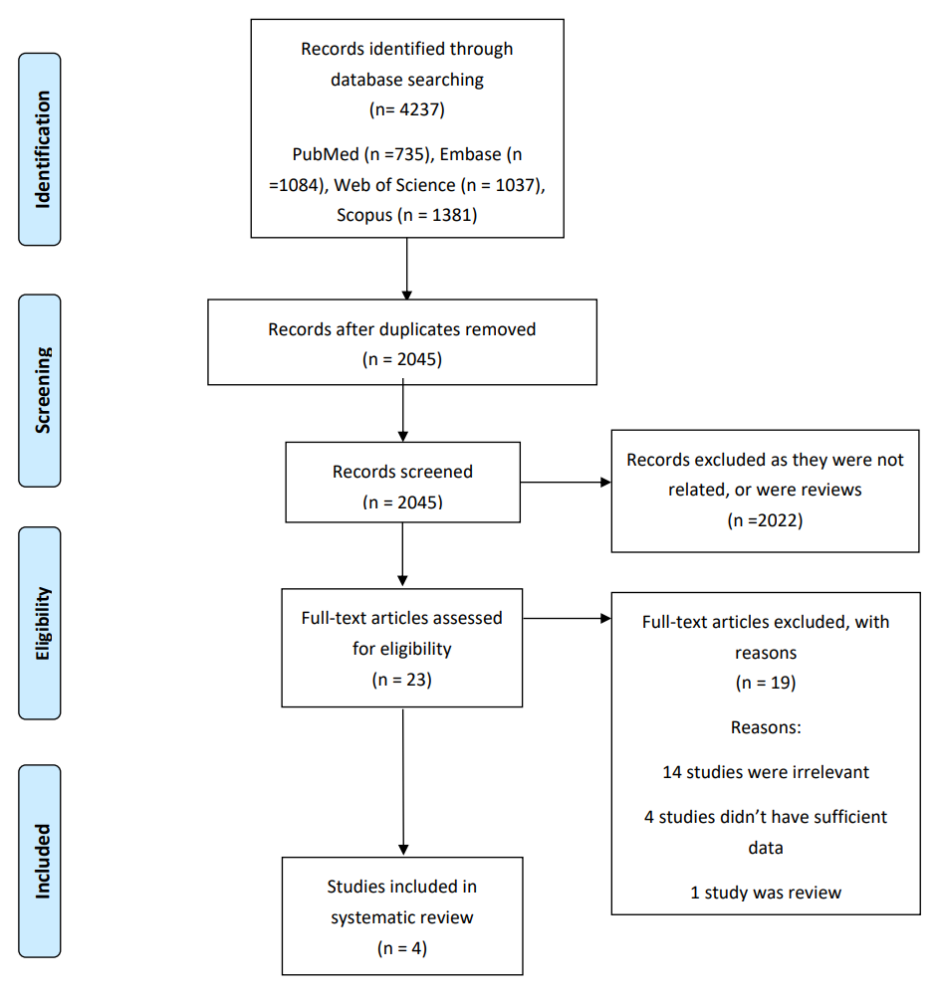
The flow chart of studies inclusion.

A literature search revealed 4237 published studies. After removing duplicates, we had 2045 records for more evaluation. Twenty-three full texts were evaluated, and four studies remained for systematic review (Figure 1).

Three studies were conducted in the United States and one in the Netherlands. The mean age of patients at the time of study varied between 11.9 and 17.9 years, and the mean age at the onset of the disease ranged between 11.5 and 14.3 years. Most included patients were female, and the NOSs of included studies were 8 or 9 (indicating a low risk of bias). The results show that median richness (the number of unique taxa identified), which was provided by two studies, was higher in controls, and the Margalef index, which was reported by one study, was higher in the control group than the case group. The results of two studies also demonstrated that median evenness indexes (taxon distribution, Shannon, Simpson) were higher in control groups, as well as PD (Faith's phylogenic diversity metric) (Table 1).

**Table 1.** Data extracted from included studies.

**Author**	**Year**	**Country**	**Study design**	**Study participants**	**Age (Year)**	**Gender**	**Race**	**Ethnicity**	**BMI**	**MS duration (Month)**	**EDSS Median (range)**	**Age at MS onset (Year)**	**Sample type**	**Richness (the number of unique taxa identified (species-level OTUs))**	**Richness (number of observed ASVs (Margalef index))**	**Richness(Chao1)**	**Evenness (Taxon distribution) Median (quartiles)**	**Evenness (Shannon)**	**Evenness (Simpson)**	**PD (Faith's phylogenic diversity metric)**	**NOS Score**
**Tremlett et al. [11]**	2016	United states	Case-control	24 Case: 15 Control: 9	Case: 11.9 ± 4.64 Control: 13.8 ± 3.19	Case: F: 8 M: 7 Control: F: 7 M: 2	Case: White:5 Non-white: 10 Control: Whit: 6 Non-white: 3	Case: Hispanic:6 Non-Hispanic: 9 Control: Hispanic:3 Non-Hispanic: 6	Case: 22.2 ± 6.21 Control: 20.9 ± 4.89	10.0 ± 6.42	2.0 (0-4.0)	11.5 ± 4.84	Stool	Median (quartiles) Case: 1452 (2089, 2975) Control: 1783 (2976, 3239)	NR		Case: 0.282 (0.319, 0.378) Control: 0.312 (0.346, 0.386)			Median (quartiles) Case: 58.1 (83.9, 107.7) Control: 69.0 (105.4, 123.9)	8/9
**Bruijstens et al. [12]**	2022	The Netherlands	Case-control	50 Case: 26 Control: 24	Median (IQR) Case: 17.3 (15.5–18.6) Control: 10.6 (6.7–14.2)	Case: F: 17 M: 9 Control: F: 15 M: 9	N/A	Case: European: 12 Non-European:14 Control: European: 17 Non-European:7	N/A	Median (IQR) 30.0 (19.3–37.8)	N/A	Median (IQR) 14.3 (13.3–15.9)	Stool	NR	NR	Median (IQR) Case: 210.14 (178.06, 255.94) Control: 231.01 (202.60, 246.09)	NR	Median(IQR) Case: 4.20 (3.86, 4.46) Control: 4.28 (3.85, 4.46)	Median(IQR) Case: 0.97 (0.958,0.98) Control: 0.969 (0.942,0.976)	NR	8/9
**Tremlett et al. [13]**	2016	United states	Case-control	35 Case: 18 Control: 17	Case: 12.5 ±4.44 Control: 13.5 ± 3.08	Case: F: 10 M:8 Control: F: 9 M: 8	Case: White: 9 Non-white: 9 Control: White: 13 Non-white: 4	Case: Hispanic: 8 Non-Hispanic: 10 Control: Hispanic: 6 Non-Hispanic: 11	Case: 22.2 ± 5.66 Control: 22.8 ± 7.10	10.6 ± 6.45 months	2.0 (0-4.0):	12.1 ± 4.66	Stool	Median (quartiles) Case: 2429 (1502-3209) Control: 2779 (1848-3095)	NR	NR	Case: 0.34 (0.29-0.38) Control: 0.35 (0.31-0.37)			Median (quartiles) Case: 94.3 (58.5-123.7) Control: 103.5 (71.6-119.1)	8/9
**Tremlett et al. [14]**	2021	US, Canada	Case-control	68 Case: 32 Control: 36	Median (IQR) Case: 22.8 (13.8-36.3) Control: 19.9 (13.2-29.9)	Case: F: 24 M: 9 Control: F: 21 M: 15	Case: White:17 Non-white: 15 Control: White:13 Non-white: 23	NR	N/A	N/A	N/A	N/A	Stool		Median (IQR) Case: 18.2 (15.9, 22.1) Control: 19.7 (16.7,22.9)	Median (quartile) Case: 216 (174-257) Control: 236 (201,260)	NR	Median (quartiles) Case: 0.682 (0.634) Control: 0.680 (0.643, 0.693)		NR	7/9

OTU: Operational taxonomic unit or OUT, NA: Not applicable, EDSS: Expanded disability status scale, ASV: Amplicon Sequence Variant, NA: Not applicable, NR: Not reported

## 4. Discussion

In recent years, growing evidence suggests that the gut microbiome, the collection of microorganisms residing in the gastrointestinal tract, may play a role in developing MS, as well as its progression [8],[13].

The gut microbiome is a diverse ecosystem of bacteria, fungi, viruses and other microorganisms that coexist in symbiosis with the human host. It performs essential functions, such as nutrient metabolism, immune modulation and protection against pathogens. Perturbations in the gut microbiome composition, known as dysbiosis, have been implicated in various autoimmune and inflammatory conditions, including MS [11],[14].

Our choice of focusing on pediatric MS patients stems from the distinct advantage they offer in investigating associations closely aligned with the original exposures and biological onset of the disease. Unlike adult MS cases, studying pediatric MS allows for a unique opportunity to explore these connections with minimal interference from a lifetime of diverse exposures, thus minimizing potential confounding effects. By concentrating on this specific population, we aim to provide a more nuanced understanding of the microbiome status during the onset of pediatric MS.

Studies investigating the link between the gut microbiome and pediatric-onset MS have focused on analyzing the microbial composition and functional profiles of patients compared to healthy controls. These studies utilize advanced sequencing techniques, such as the 16S rRNA gene and meta-genomic sequencing, to identify specific microbial taxa and functional pathways associated with the disease [15],[16].

Researchers in 2017 analyzed the gut microbiome of pediatric-onset MS patients and found alterations in microbial community structure in the case group compared to healthy controls, as well as pediatrics with other neurological disorders. They observed reduced levels of beneficial bacteria such as Prevotella, and Veillonella in the MS group, while certain potentially pathogenic bacteria like Parabacteroides distasonis were more abundant [16].

Other researchers published a study in 2016 and investigated the fecal and oral microbiome alteration of pediatric-onset MS patients and distinct microbial profiles compared to the healthy controls. They reported decreased microbial diversity and alterations in specific bacterial taxa, including increased levels of Akkermansia muciniphila and decreased levels of Blautia and Ruminococcus [15].

The existing body of research, encompassing both pediatric and adult cohorts of MS patients, consistently reveals a noteworthy reduction in gut microbiota and its diversity when juxtaposed with demographically matched healthy counterparts [8],[11]–[14],[17]. This convergence of findings underscores the potential relevance of the gut microbiome in the context of MS pathology.

Within the scope of our systematic review, incorporating a comprehensive analysis of four pertinent studies, a recurrent theme emerges with respect to diminished richness in MS cases as opposed to their healthy counterparts. Richness, defined by various metrics such as the number of unique taxa identified at the species level (species-level OTUs), the number of observed Amplicon Sequence Variants (ASVs) (Margalef index) and Richness (Chao1), consistently manifests as quantitatively reduced in the microbiota of individuals afflicted with MS [11]–[14]. Although this observation is recurrent across the examined studies, the establishment of statistical significance necessitates a meta-analytical approach that aggregates data across diverse investigations.

Furthermore, the exploration of evenness, as assessed through taxon distribution [11],[13], Shannons Evenness [12],[14] and Simpson's Evenness [12], was conducted in a subset of studies. These parameters collectively elucidate the distributional uniformity of microbial taxa within the microbiome. Despite the limited number of studies focusing on these aspects, the available evidence suggests a semblance of consistency in the evenness of microbial composition between MS cases and healthy controls.

These studies, and others, provide preliminary evidence of a potential association between the gut microbiome and pediatric-onset MS [16],[18]. However, the underlying mechanisms driving this association are not yet fully understood. Several mechanisms have been proposed:

*Modulation of the immune system:* The gut microbiome interacts with the host immune system and helps regulate immune responses. Dysbiosis may disrupt this delicate balance, leading to immune dysregulation and increased susceptibility to autoimmune diseases like MS. Certain gut bacteria produce metabolites, such as short-chain fatty acids, that influence immune cell function and inflammation [12],[18].

*Intestinal barrier function:* The gut microbiome plays a role in maintaining the integrity of the intestinal barrier, which prevents the leakage of harmful substances into the bloodstream. Disruptions in the gut microbiome can compromise the barrier function, allowing the translocation of microbial products, and triggering immune responses [11],[13],[14].

*Molecular mimicry:* Molecular mimicry occurs when microbial antigens resemble host antigens, leading to cross-reactivity of the immune system against self-antigens. This phenomenon has been implicated in the pathogenesis of autoimmune diseases, including MS. Certain gut bacteria may possess antigens that resemble components of the central nervous system, potentially triggering autoimmune responses [4],[6],[8],[18].

*Metabolite production:* The gut microbiome produces various metabolites that can influence immune function and neuroinflammation. For example, certain gut bacteria generate metabolites like butyrate and propionate, which have anti-inflammatory properties and can modulate immune cell function [16],[18].

It is important to note that the gut microbiome is highly individualized, and variations can occur based on factors such as diet, geography, genetics and early-life exposures. Additionally, the gut microbiome is not limited to bacteria alone and also includes other microorganisms like fungi and viruses, collectively referred to as the mycobiome and virome, respectively. Research into the role of these components in pediatric MS is emerging and warrants further investigation [14].

While the association between the gut microbiome and pediatric MS shows promise, it is crucial to interpret the findings with caution. The studies conducted so far have small sample sizes, and more extensive longitudinal studies are needed to establish causality, determine the specific microbial signatures associated with pediatric MS and explore the potential for microbiome-based interventions as part of MS management.

This systematic review has some strengths. First, it is the first study in this field. Second, we gathered all evidences. Third, our search was systematic.

It also has some limitations. First, the number of studies was limited. Second, we could not perform meta-analysis. In light of these observations, it is imperative to underscore the preliminary nature of these findings, which necessitates a more comprehensive synthesis through meta-analytical methods. The establishment of statistical significance, robust generalization, and a deeper understanding of the nuanced dynamics underlying the observed alterations in gut microbiota parameters in MS necessitate further investigations. As the field progresses, a heightened emphasis on meta-analytical endeavors will contribute to refining our comprehension of the intricacies surrounding the relationship between the gut microbiome and the onset of MS.

## 5. Conclusion

The result of this systematic review showed disruption of the microbiota-immune balance in pediatric-onset MS cases.
